# Reports on Hospitals of the United Kingdom

**Published:** 1914-06-13

**Authors:** Henry Burdett


### REPORTS ON

### Hospitals of the United Kingdom.

By SIR HENRY BURDETT, K.C.B., K.C.V.O.

SERIES III.

### KENT COUNTY OPHTHALMIC HOSPITAL, MAIDSTONE.

THIS hospital was instituted in 1847, and provides treatment for patients suffering from diseases of the eye, ear, nose, and throat. It contains thirty-nine beds.

On entering, one is faced by an imposing staircase, which leads to the women's wards, those for the men being situated on the ground floor. The chief features of the hospital are two very large and attractiive double-windowed day rooms, one for men and one for women, and an addition to the out-patients' department which provides accommodation for the treatment of an unusually large number of adenoid cases, of which this hospital seems to have the monopoly so far as Maidstone and the district are concerned. It is manifest to us that as the majority of adenoid cases appear to be sent to this type of hospital by the County Education Authorities, the authorities here and everywhere where this practice prevails should agree to defray the cost of treating these cases and also to pay the cost of the medical service.

The most has been made of the garden, of which the attractiveness has been increased by the addition of a walk extending its whole length, and over this walk have been trained a number of crimson and other ramblers, which have a most pleasing effect during the time the roses are in bloom.

The Matron, Miss Gertrude Brown, has been connected with this hospital for a number of years, and her services and skilled co-operation are specially acknowledged by the honorary surgeons in their report to the hospital. We note with satisfaction that the enlargement of the out-patients' department was accompanied by the appointment of an out-patient sister to take charge of the work. Included in the additions are a consulting-room, dark-room, the sight-testing range for eye patients, and a casualty operation theatre. The total cost was some £1,000.

This hospital contains a chapel, which is an unusual addition to a special hospital of its type.

The financial position of the hospital would appear from the accounts to be reasonably satisfactory. That a revenue of £1,025 a year should be derived from investments, out of a total income of £2,120, is a notable fact due, we believe, to the circumstance that this hospital has been established upwards of sixty years. Again, it is surprising that no payments appear to have been made by the patients, an omission which, having regard to the nature and character of the work done, demands the attention of the committee. The hospital has, then, in this omission to take payments from its patients, a large reserve of income available, for it may clearly follow the example of other special hospitals by making a charge for both out-patients' and in-patients' treatment under proper regulations which exhibit a due regard to the circumstances of each patient.

